# Comprehensive patient education and counselling for non-communicable diseases in primary care, Western Cape

**DOI:** 10.4102/safp.v65i1.5634

**Published:** 2023-02-10

**Authors:** Robert J. Mash, Joleen Cairncross

**Affiliations:** 1 Division of Family Medicine and Primary Care, Faculty of Medicine and Health Sciences, Stellenbosch University, Cape Town, South Africa

**Keywords:** noncommunicable diseases, diabetes, patient education, counselling, primary care, behaviour change

## Abstract

**Background:**

Treatment of non-communicable diseases (NCD) requires patient education and counselling (PEC). Initiatives have focused on Group Empowerment and Training (GREAT) for diabetes and Brief behaviour change counselling (BBCC). However, the implementation of comprehensive PEC in primary care remains a challenge. The aim of this study was to explore how such PEC could be implemented.

**Methods:**

This was a descriptive, exploratory, qualitative study at the end of the first year of a participatory action research project to implement comprehensive PEC for NCDs at two primary care facilities in the Western Cape. Focus group interviews were held with healthcare workers and reports from co-operative inquiry group meetings were used as qualitative data.

**Results:**

Staff were trained in GREAT for diabetes and BBCC. There were problems with training appropriate staff and sufficient numbers and a need for ongoing support. Implementation was limited by poor internal sharing of information, staff turnover and leave, rotation of staff, lack of space and fears of disrupting the efficiency of service delivery. Facilities had to embed the initiatives into appointment systems and fast track patients who attended GREAT. For those patients that were exposed to PEC, there were reported benefits.

**Conclusion:**

Group empowerment was feasible to introduce, while BBCC was more challenging as it required extra time in the consultation.

**Contribution:**

Implementation of PEC requires alternative approaches that do not extend consultations (such as GREAT and maybe digital solutions) as well as commitment to facility organisation for PEC from managers.

## Introduction

Non-communicable diseases (NCDs) are one of the major health and development challenges of the 21st century, not only because of the human suffering they cause but also because of the harm they inflict on the socioeconomic fabric of countries, particularly low- and middle-income countries.^1^ As the leading cause of death globally, NCDs are responsible for 40 million (or 70% of) deaths annually. Each year, 15m people die from an NCD between the ages of 30 and 69 years; over 80% of these ‘premature’ deaths occur in low- and middle-income countries.^1^ Sub-Saharan Africa is undergoing a rapid epidemiological transition as the rising burden of NCDs collides with epidemics of existing chronic infectious diseases such as HIV and tuberculosis.^2^

In South Africa, diabetes is now the leading cause of death in women and the second leading cause overall.^3^ Non-communicable diseases account for 51% of all deaths, including 19% from cardio and cerebrovascular causes, 8% from diabetes and chronic kidney disease, 10% from cancers and 5% from respiratory diseases. In the Western Cape from 2009 to 2013, the mortality surveillance trends attributed 60% of deaths to NCDs (cancers, cardiovascular, diabetes and other causes) in both males and females.^4^

The recently published National Strategic Plan for NCDs targets five NCDs (cardiovascular, chronic respiratory, cancer, diabetes as well as mental and neurological disorders) and five risk factors (unhealthy diet, tobacco use, harmful alcohol use, physical inactivity and air pollution).^5^ This is referred to as the 5 × 5 strategy. A cascading strategy of 90-60-50 is recommended for diabetes and hypertension^5^:

- 90% of all adults will know if they have raised blood pressure or glucose.
- 60% of those identified will receive intervention.
- 50% of those receiving an intervention are controlled.

Goal 3 focuses on ensuring that people living with NCDs receive integrated, people-centred health services to prevent complications and control the disease. One of the key strategic objectives is to implement appropriate communication, health literacy and behavioural initiatives.

Previous work by researchers within the Chronic Disease Initiative for Africa (CDIA) in Cape Town, South Africa, has developed most of the components of a comprehensive approach to patient education and counselling (PEC). A pragmatic clustered randomised control trial demonstrated that group diabetes education delivered by health promoters (mid-level workers) in a guiding style (derived from motivational interviewing) is a cost-effective intervention in the setting.^6^ Training and resource materials for group diabetes education were developed in this project, and similar materials for asthma and chronic obstructive pulmonary disease (COPD) were developed in the Asthma Guideline Implementation Project.^7^ A previous quasi-experimental study also showed that individual brief behaviour change counselling (BBCC) using the 5As best practice guideline (Ask, Alert, Assess, Assist and Arrange) with a guiding style is effective in helping pregnant mothers stop or reduce tobacco smoking.^8^ A training module for primary care providers and patient education materials has been developed for this programme (www.ichange4health.co.za).^9^ Training primary care providers to deliver BBCC can be effective, at least in the short term.^10^ However, training alone might not be enough to ensure implementation of BBCC at the coal face where it needs to be delivered.

Although it is clear from the evidence that training can enable primary care providers to deliver behaviour change counselling effectively, delivering this counselling in our clinical environment could be challenging.^10^ Community health centres (CHCs) are challenged by high volumes of patients with NCDs who experience long waiting times for brief and often unsatisfactory encounters with their primary care providers.^4^ Health services are under pressure to improve their efficiency and throughput of patients as well as the quality of care received. Care for NCDs is well supported by the Practical Approach to Care Kit (PACK) guide, which provides an integrated approach to managing chronic conditions.^11^ Such an approach requires attention to medication as well as empowering patients to understand and self-manage their condition. Attention has been given to the technical quality of care through regular audits and suggests services are prescribing suitable medication.^12^ However, there appears to be significant clinical inertia in the system as there is little change to patient’s management despite evidence that they are poorly controlled.^13^ Although these audits record high levels of PEC, this is often ticked off as a result of talks in the waiting room or as a result of brief directive advice giving. Evidence suggest that patients have low health literacy, are poorly informed about their conditions, and not empowered in self-care management.^14^ There is a need to explore ways in which a more comprehensive patient education and counselling can support patient self-management and improved control.

This study evaluates how two primary care facilities in the Western Cape attempted to implement a more comprehensive PEC. This evaluation was conducted at the end of the first year in 2020. Unfortunately, the project was disrupted by the coronavirus disease 2019 (COVID-19) pandemic at the beginning of 2020. The main aim of this study was to explore what was learnt during this year on how to implement a more comprehensive PEC in one urban and one rural primary care facility within the public sector of the Western Cape, South Africa.

## Methods

### Study design

This was an exploratory descriptive qualitative study using focus group interviews (FGIs) and reports from co-operative inquiry group (CIG) meetings.

### Setting

Two community day centres (CDCs) were selected for the study as both showed interest in exploring how to implement a more comprehensive PEC for NCDs. The one CDC was in the Metropolitan Health Services and situated in the Northern-Tygerberg substructure of Cape Town. The other CDC was in the Rural Health Services and situated in Stellenbosch a rural town within the Cape Winelands District.

Both CDCs had a multidisciplinary team involved in the delivery of care for people with NCDs. The teams included clinical nurse practitioners (CNPs), medical officers, pharmacists, health promotion officers and other nurses. In the urban CDC, they also had a dietician and a family physician as part of the team. These CDCs offered chronic care to people with any NCDs, including diabetes, hypertension, asthma and COPD.

The urban CDC served uninsured communities on the Cape Flats who lived in low-cost housing and spoke either Afrikaans or English. The rural CDC served uninsured communities in and around the town of Stellenbosch included farming areas. This population mostly spoke Afrikaans.

### The co-operative inquiry groups

Implementation of a more comprehensive PEC for NCDs at both sites was driven by participatory action research. A CIG^15^ was established at each site that consisted of the key staff members involved in chronic care and the facility management. The groups were meant to participate in action and reflection over a 2-year period. During 2019, each group had four formal meetings, and a brief summary of each meeting was made by the research coordinator (J.C.). These eight reports at the end of year-1 were used as qualitative data in this study. The second year of the co-operative inquiry did not happen because of the advent of the COVID-19 pandemic.

### Key actions taken by the co-operative inquiry groups

At both sites, the CIGs focused on two key interventions: firstly, the introduction of Group Empowerment and Training (GREAT) for people with diabetes, and secondly, the introduction of BBCC for all clinicians that consulted people with NCDs.

Key staff at each facility were trained in facilitating GREAT for diabetes with groups of patients. These staff might be nurses, health promoters, medical officers, pharmacists or dieticians. A 3-day training introduced them to the structure of the group sessions, the essential communication skills for a guiding style and the resource materials used in the sessions. Training was conducted by the research coordinator (J.C.). Group Empowerment and Training for diabetes is more fully described in other publications.^16,17^

All clinicians were invited to attend a 1-day training in BBCC that was also conducted by the research coordinator (J.C.). This model of BBCC combined the 5 As (Ask, Alert, Assess, Assist and Arrange) with a guiding style and has been more fully described in other publications.^9^

### Selection of participants

All staff members involved in the implementation of a more comprehensive PEC at each site were invited to a FGI at the end of year one (February 2020). This excluded the facility managers to avoid social desirability bias. Managers also gave feedback within the CIGs. Further interviews were precluded as all research activities were suspended with the advent of the COVID-19 pandemic.

### Data collection

An independent research assistant with experience in qualitative interviewing, and who was not part of the co-operative inquiry or implementation, conducted the interviews. The researcher was a nurse by background with experience of working in similar settings. An interview guide was used to explore the experience of staff during the first year of the co-operative inquiry. Key topics that were explored included the appropriateness of changes made to service delivery, any training received, the feasibility of implementing change and the fidelity of such changes to the training received, as well as the sustainability of any changes.

Focus group interviews were conducted in English at the primary care facilities in a private room. English is the usual language of organisational communication. The interviews were recorded digitally and lasted between 30 min and 60 min.

### Data analysis

The FGIs were transcribed verbatim and checked for accuracy. Analysis was performed by the researcher (RM) with the help of Atlas-ti software (Version 8). In addition, the eight reports from the CIGs were included in the analysis. The analysis followed the steps of the framework method^18^:

- Familiarisation: Reading the transcripts to become familiar with the data.
- Coding index: Identifying codes from the data and organising them into categories.
- Coding: Coding the transcripts.
- Charting: Creating reports where all data on the same code or category is brought together.
- Interpretation: Interpreting the reports for key themes and sub-themes.

## Findings

### Respondents

Two FGIs were held, one at each of the sites. At the urban facility, the FGI included the dietician, three CNPs, an enrolled nursing assistant (ENA) and an administrative clerk. At the rural facility, the FGI included the pharmacist, a CNP, a health promotion officer (HPO) and a medical officer (MO).

### Adoption and appropriateness

Respondents recognised that NCDs were a huge contributor to the burden of disease and that PEC was part of their work. They felt that the group education was an appropriate strategy and that BBCC should easily be integrated into consultations:

‘We have lots of patients that have these chronic diseases … the COPD/asthma group just started last week also. So I understand that’s also going well. But that’s … yeah. It’s common in the community so we need this.’ (FGI Urban CHC)

### Feasibility of implementing patient education and counselling

#### Issues with training

Respondents were very positive about the training and how they had learnt new competencies:

‘To me, it was brilliant, it was good because it opened our minds up to the thought that what we were actually doing was wrong. I won’t say it was wrong it worked for us because we didn’t know, we didn’t know, but after we went to the BBCC, yoh we were all talking yoh and we actually laughed when we spoke to her and she said …’ (FGI Rural CHC)

However, they thought more staff involved in consulting patients with chronic diseases should be trained, to make PEC more sustainable. This would mean that there would be more people available to cover for leave and to share the burden of facilitating groups:

‘We definitely need more people to be trained. Also, it can become very exhausting when like you’re driving the group all the time and you have to now … I have to book more patients and so on.’ (FGI Urban CHC)

In some instances, staff not involved in consulting patients were trained, and it was not clear how this benefited them. For example, an enrolled nurse in the preparation room was not able to perform BBCC but rather identified people that would benefit from group education or BBCC. A clerk in reception was trained in BBCC and was not able to implement it although appreciated learning the communication skills. At the urban CDC, the nurse practitioners trained in BBCC were working in the general adult clinic and not the club for NCDs. This meant they only saw people being linked back into care or were newly diagnosed. At the rural CDC, the nurse practitioner seeing children found it difficult to implement BBCC. Selecting the right people for the specific training was therefore important:

‘We don’t see a lot of … because most of the people … Our chronic patients are seen at the club. So we only see those that defaulted. Stable ones and those new ones that they come with other ailments and we pick up they have got high blood and diabetes.’ (FGI Urban CHC)

‘Other healthcare workers trained in BBCC, may not have been appropriately selected as they don’t offer individual counselling with patients, nor are they allocated to the area where chronic patients with the NCDs being investigated are attending. This included clerk, ENAs, EN and the HPOs.’ (CIG Urban, 03 December 2019)

Staff felt that there was good follow-up and support after training via meetings and WhatsApp groups, but consistent reminders were needed to ensure implementation continued.

#### Implementation of the new skills

In both settings, it took several months to actually implement group sessions after the training. This might have been because the trained facilitators lacked confidence and introducing new components into service delivery was difficult. In some cases, participants missed the training session on how to implement group education in the facility. The workload, time constraints and low staffing levels were given as reasons for not implementing the group sessions. Staff did not want to disrupt service delivery and in some cases tried to apply what they had learnt within the existing system. For example, the health promotion officer at one site started to use pieces of the group session’s resources in her talks to the waiting room or tried to include aspects of BBCC when taking their observations. The impression was that the research coordinator. (J.C.) needed to be persistent in supporting actual implementation of the group sessions:

‘They were trained in April and had not presented any sessions until November and felt “rusty” and may have forgotten how to present the session.’ (CIG Rural, 03 December 2019)

‘Staff workload, staff absenteeism (training, outreach, leave and illness) and time constraints were the biggest challenges. New referrals of patients not formally booked at the clinic was also mentioned.’ (CIG Rural, 25 October 2019)

‘CIG participants agreed they were not implementing diabetes group education training as it was intended to be used. On the last day of their training they missed the planning session on how to implement diabetes group education in the facility which may have been a contributing factor.’ (CIG Rural, 30/8/2019)

Staff were reluctant to do anything that would interfere with service delivery. The impression was that this meant the efficient flow of patients through the facility and managing to get through the workload by the end of the day. The introduction of a more effective PEC was perceived as a threat to service delivery, rather than as a means of improving the quality:

‘As part of routine health talks, not the structured group diabetes education sessions nor BBCC, the HPO would conduct this in the patient waiting area. The HPO would deliver the talk on a specific health theme and provide patients with these resources. Due to the Club room being occupied daily, it is not possible for the HPO to conduct health talks in the Club room. Other staff members are also using the room daily and it would interfere with service delivery. However health educational videos are played in the Club room.’ (CIG Urban, 28/8/2019)

#### Experience of patient education and counselling

Participants were very positive about the resource materials used in the group sessions and topics covered. They also reported that they had shifted in terms of their communication style and that PEC was now more interactive and collaborative.

Participants reported that their messages and education about NCDs were more consistent as a result of attending the same training. For some, these changes became integrated into how they now consulted patients:

‘Yeah so from the group I think it’s a very nice content. It covers all the important topics and I like the style of counselling. It’s very interactive with the patients as well and they enjoy that yeah.’ (FGI Urban CHC)

‘The clients feel more open to talk because they don’t feel like they’re being pressurized to doing things or saying things or they’re not too scared to say things, they feel more open to discuss things, so I think the – it’s nice.’ (FGI Rural CHC)

‘And the patient actually opens up more now because it’s not like we’re fighting with them cause there are reasons why they sometimes default or why they’re not taking their medication right but before we used to say ja but if you don’t do this, this is going to happen and that, now that we’ve changed the counseling style and the approach, it’s working out better.’ (FGI Rural CHC)

They noted that in the groups, patients also motivated each other, shared feasible ways of changing behaviour and were less resistant to change. There were one or two problems with dominating personalities that had to be managed:

‘I also preferred the group ones because then they can like help each other and like encourage each other on what to eat and what to do and what this one is doing, what that one is doing so, yes, the group one is very nice, yes, because you are like a small bond.’ (FGI Rural CHC)

BBCC took longer to perform than in the training, especially when it was a new competency. This could add too much time to the consultations and made it difficult for some clinicians to perform BBCC:

‘I’m the only Medical Officer at the day hospital, so it’s very difficult because I don’t only do “chronics”, I do “acutes”, I do other referrals as well, so sometimes it is very difficult to implement BBCC when you have a fully booked day, you’ve got nurses coming into your room, I also have a session at the hospital in the morning so everything is like a rush, so it is a bit difficult, but I do like touch on this and touch on that and talk to them about their diet and talk to them about medication.’ (FGI Rural CHC)

There were some concerns about literacy and language barriers with the materials given to take home although they saw that these could reinforce behaviour change. These new materials were combined with existing materials in the consultations although the distribution of the new materials to all clinicians took some time:

‘The MO was not using the “Nutrition Issue” when conducting BBCC addressing eating healthy, but the existing clinic resources (handout) on dietary advice. The four patient issues (Nutrition Issue, Physical Activity Issue, Smoking Issue, Alcohol Issue) was not available in her consultation room.’ (CIG Rural, 03 December 2019)

‘The Adult Road To Health Cards (ARTHC) and the patient education leaflets (Nutrition Issue, Physical Activity Issue, Alcohol Issue and Smoking Issue, previously delivered a few months ago, are not being used during patient consultations. CIG members were not sure where this is being stored in the storeroom.’ (CIG Urban, 03 December 2019)

#### Accommodation of access to patient education and counselling

At the urban CDC, they initially attempted to get patients to attend on separate dates for the group sessions, but attendance was poor as they did not want to make an additional visit to the health centre:

‘Patients however did not attend the booked dates for sessions. Contributing factors to non-attendance were having the diabetes group education appointment date not on the same date as for a consultation nor medication collection. In the case of patients booked for medication collection, they may send someone else to collect their medication. Employed patients did not want to attend the sessions as it implied another day of sick leave. Following the non-attendance, patients were then referred from the “Club” room by doctors and clinical nurse practitioners [CNPs]. Majority of referrals came from the doctors.’ (CIG Urban, 16 October 2019)

At both facilities, they thought through how to give appointments so that patients received the next group session at their routine visit. Although both facilities noted that many patients did not keep their appointments and not everyone completed all the group sessions. At the urban CDC, the facilitator phoned the non-attenders to remind them and find out why they did not come. Patients reported that they forgot or had made a different appointment:

‘Okay so I have a book, the diary that I put the sticker in. And then if the patient has to come back for the doctor then we try to book it on the same day at the help desk. So then I will maybe … Maybe in a months’ time then I will have the next session so that it’s the same day as the doctor so that they come for the doctor and the session yeah. Because patients are working and we don’t want to keep them also out of work too much.’ (FGI Urban CHC)

At the urban CDC, patients were fast tracked after attending the group through the preparation room, consultation and pharmacy so that they were not penalised. At the rural CDC, patients had to come for the group session before the day started as staff did not want to disrupt service delivery, and this restricted the people that could attend to those that lived nearby:

‘Because what we had to do is, we had to now start the sessions, the group sessions, like before we started working, so from seven o’clock we would start and do the observations and do everything. Start the group session, so by nine o’clock when the normal clinic duty starts, we were done with the group session.’ (FGI Rural CHC)

‘Because we explain it to them when we give them the date and the time. So then when they’re done with the session then they … if they have to see the doctor they get fast tracked. So they go to prep room, prep sister fast tracks them. Then they go to the doctor. So they get fast tracked by the doctor. Then they go to pharmacy also for being fast tracked. So they don’t spend the whole day in the clinic. So that’s like their incentive for coming.’ (FGI Urban CHC)

#### Relationship of brief behaviour change counselling to group empowerment and training

Brief behaviour change counselling and group empowerment and training group sessions were seen as complementary. In the urban CDC, they felt that BBCC increased the confidence of patients to participate in the group sessions. While in the rural CDC, they felt that the group sessions prepared patients for more targeted individual conversations with the clinician, because they already understood the background of their disease and self-management:

‘The patient is free to talk of whatever problem there is [*in BBCC*] and [*that*] he/she encounters in this. Not like in the group where some of the patients feel shy to say whatever they feel like, feel free to say in the group. So I like it that way. By the time the patient goes to the group she’s prepared, she knows what kind of question to ask the group or the person in charge of the group.’ (FGI Urban CHC)

‘And the nice thing with the nice thing is if the people attend the group sessions by the time they get to the doctor … Then they’re informed about everything … We talk the same language.’ (FGI Rural CHC)

#### Staffing levels and turnover

Staffing levels and turnover influenced the ability to offer PEC. In the urban CDC, staff turnover meant that many of those trained were no longer available, while in the rural CDC, staffing levels made it difficult to integrate PEC into the normal services. For example, there was only one medical officer, with many other responsibilities, who struggled to offer BBCC and get through the list of booked patients. Having key staff on leave made it impossible to run the group education and the facility felt that they had to plan for sessions months in advance when no one was on leave:

‘I’m the only CNP. We were the two of us. The one that I did it with is the one that left, they’re working in the other facility so I’m the only one right now that did the BBCC.’ (FGI Urban CHC)

‘Four staff members were booked for BBCC training in November and only one attended due to unavailability of locum healthcare workers to replace them at the clinic. Several staff members trained in BBCC are not working at the clinic anymore. This influences if patients are receiving BBCC as part of implementation. The MO and Family Physician have left employment. One of the CNPs will start new employment.’ (CIG Urban, 03 December 2019)

#### Space for group education

Space was an issue at both sites. In the urban CDC, they used the dietician’s office but could only accommodate six to eight people. In the rural CDC, they used the resuscitation area in the emergency room, but of course this had to be vacated in an emergency, and many services were competing for space such as visiting therapists and consultants. Health centres were not designed with the need for group education in mind. In summer, the groups could meet outside in the courtyard or under a gazebo:

‘Group discussion, if there’s an emergency and there’s a group discussion, so emergency takes preference and we do have a lot of outreaches as well from the physios, I mean from the OTs and the speech therapists and the psychologists, psychiatric centers and stuff like that.’ (FGI Rural CHC)

#### Communication within facilities

The CIG reported on many problems with internal communications, which meant not all staff understood what was being implemented as well as who and how to refer patients. At the urban CDC, those doing eye screening and optometry were unaware of the initiatives in PEC. The initiative was not always discussed in staff meetings. Referral forms for the group sessions were created but not utilised. The introduction of WhatsApp groups did facilitate communication between the implementers and the rest of the staff:

‘Group diabetes session dates have been posted on the facilities WhatsApp group. All clinical staff members are on the WhatsApp group. More effective way to inform all clinical staff members on session dates. However, the dietician suggested frequently reminding clinical staff on the WhatsApp group would be more helpful and increase patient referrals.’ (CIG Urban, 03 December 2019)

‘The Operational Manager expressed doubts that everyone at the facility were aware of the diabetes group education implementation. The protocol at the facility is monthly clinical meetings where all clinical service issues are discussed. There’s not been a meeting where this was discussed.’ (CIG Urban, 16 October 2019)

‘The individual HCW referral document, from the last CIG meeting, where the HPO would collect the list of patient referrals from each HCW had not been implemented. The Dietician was also not aware of the document as she was not at the last meeting.’ (CIG Urban 16/10/2019)

#### Documenting patient education and counselling

At the rural CDC, the facility manager introduced new stationery to document counselling in more detail, but staff remained uncertain how to best document BBCC in the medical record:

‘The two CNPs and MO found it challenging to conduct a consultation and document the “5A’s” with a bit of detail in patient folders.’ (CIG Rural 3/12/2019)

‘Recordkeeping has changed, CIG members that attended the training are also documenting if they’ve provided any counselling. At future consultations, the next HCW can review the patient notes and continue with counselling.’ (CIG Urban, 28 August 2019)

Stickers were used on the folders to identify patients that had attended the group sessions:

‘Makeshift stickers [*unmarked patient stickers*] developed by the MO were placed on the outside of patient folders and used to document attendance and follow-up dates of sessions. This was also used as identification at the pharmacy that the patient should be “fast tracked”.’ (CIG Urban, 16 October 2019)

### Fidelity to the training

Some participants were unsure if they were practicing BBCC correctly and were looking forward to feedback on what they were doing. One nurse working in the preparation room sounded like she had returned to a more directive style of education:

‘Once I start prepping the patient [*and*] I see that the HGT [*blood glucose*] is high then I also must start talking to the patient, tell him what did you eat, you mustn’t eat that, you mustn’t eat that and like that.’ (FGI Urban CHC)

At the urban CDC, they combined the group sessions into 2 days rather than 4 to make it easier for patients to attend all the sessions. Although this also made the group interaction quite long and tiring:

‘Also because it’s four sessions and like I mentioned a lot of patients are working as well. We decided to have two sessions on one date. So we only meet with the patients twice.’ (FGI Urban CHC)

Facilitators involved other staff members in specific sessions, such as the occupational therapist for foot care and the pharmacist for medication. Rather than just discussing foot care, they also actually screened people’s feet with the use of the monofilament:

‘Patients are more engaged on the session where footcare is discussed and the HPO does the mono-filament test, as well as a general foot inspection.’ (CIG Urban, 03 December 2019)

Although the health promoters were the focus for facilitating group sessions, it was the dieticians who became the main champions of implementing and facilitating sessions:

‘The HPO have found patients excited to attend the sessions, but her interaction has been limited as facilitator conducting sessions with patients. She had other work duties as the HPO which prevented her from facilitating sessions on a regular basis.’ (CIG Urban, 03 December 2019)

‘The dietician, mainly presents the diabetes group education sessions, is also not trained in BBCC to apply this during individual consultations.’ (CIG Urban, 03 December 2019)

One pharmacist saw the value of the communication skills learnt in BBCC to engaging patients at the pharmacy and trained his staff in knowledge exchange:

‘The pharmacist is using his new communication skills when sharing information with patients while dispensing their medication. He is following the “Elicit-Provide-Elicit” communication skill to enquire what patients already know or want to know. The pharmacist is applying it when discussing changes in patients’ medication, different packaging of medication […] ensures patients know they are still receiving the correct medication, and side-effects of medication.’ (CIG Rural, 30 August 2019)

### Effects of patient education and counselling

Participants gave a number of anecdotes of patients whose control significantly improved after attending group education. Patients’ knowledge and understanding improved and they made lifestyle changes even after the first session:

‘The HPO has observed a change in patients. Patients have a better understanding of diabetes and not seeing the diagnosis as the “end of the world”. They’ve also realised they don’t have to make difficult lifestyle changes in managing their diabetes. Patients are realising it’s possible to live a normal life by just making specific lifestyle changes.’ (CIG Rural, 30 August 2019)

In some, the improvement in glycaemic control was dramatic, and they could even defer starting insulin for longer:

‘Like last year I had a guy with weird HbA1cs that was really high, not last year, it was when the other CNP was there and then she did the sessions, and we had talks and stuff like that, … and then he came to me eventually for his results and his HbA1c dropped drastically and it became normal and he so proud of it that he like went on in the clinic, shouting in the corridors about his and he was still he’s doing very well.’ (FGI Rural CHC, 25 October 2019)

‘With HGT, even their medication. Some … like one lady had to go on. She was possibly going to go on insulin and she came back the next session and she said the doctor didn’t start on the insulin you know.’ (FGI Urban CHC)

Brief behaviour change counselling was also seen to be effective in changing behaviour and even adaptations of the approach in other situations, such as the pharmacy:

‘Pharmacy staff continue to use the different style of communicating with patients [*guiding style*] as the Pharmacist taught them. Specifically they have changed how to counsel patients on medication. Patients resistant to changing their medication, accept the change after the counselling.’ (CIG Rural, 25/10/2019)

‘In one case where the patient was resistant to change, the HPO continued to use the guiding style and provided the patient with the Smoking Issue. At the next visit, the patient had cut down on her smoking after having read the information in the Smoking Issue.’ (CIG Rural, 25 October 2019)

### Sustainability

Respondents suggested a variety of future topics that might also be covered in group sessions, such as asthma, COPD, hypertension, osteoarthritis and reproductive health (family planning and cervical cancer screening). The asthma and COPD materials were piloted at both sites. The urban CDC was already planning for these other types of groups:

‘It made it easier for me also with my chronic patients. So now I group them. I group them into the diabetes group. I made my own hypertension-cholesterol group now instead of seeing one on one because it can become very tiring.’ (FGI Urban CHC)

Sustainability of the current initiatives was, however, not certain. This was because of the ever-changing staff profile as a result of rotations, resignations and leave and the difficulty with orientating and training new staff members. There were also other priorities demanding attention, such as implementation of community-orientated primary care. Sustainability was probably dependent on this being a priority in the minds of the operational and facility managers:

‘The Operational Manager was concerned of the sustainability of diabetes group education and BBCC for a number of reasons. Not all staff are trained and staff rotate after a few months, increased workload, staff absenteeism, one of the chronic CNPs are also taken from their work area to help with walk-in patients to ensure few patients are turned away and the facility must also implement Community Oriented Primary Care (COPC). However the Operational Manager believes with proper planning and good communication comprehensive PEC can be implemented.’ (CIG Urban, 16 October 2019)

The dietician at the urban CDC was trained to train future facilitators of GREAT for diabetes, and this provided considerable in-house expertise that could support sustainability:

‘During December 2019, the dietician completed the “Trainer-of-Facilitators” in diabetes group education. The dietician can now train additional healthcare providers to facilitate diabetes group education sessions.’ (CIG Urban, 26 February 2020)

## Discussion

### Summary of key findings

**FIGURE 1:**
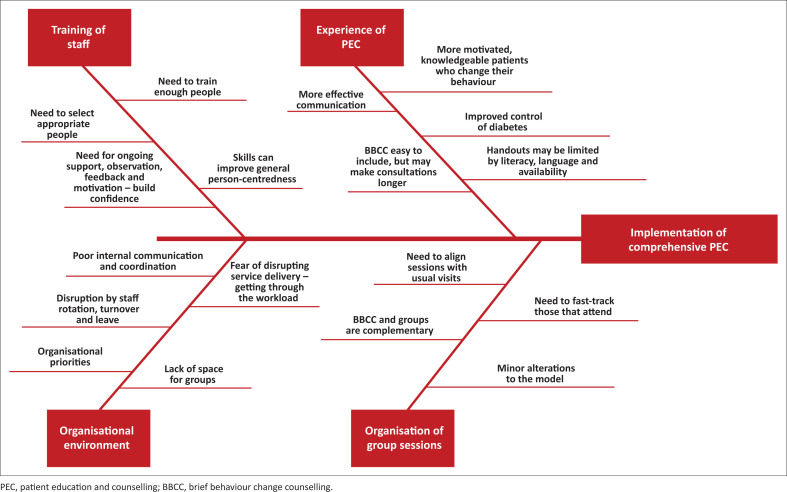
Summary of key findings.

The key findings that relate to the implementation of a more comprehensive PEC are summarised in Figure 1. The overwhelming impression was that staff were struggling with a high workload and limited human resources that were stretched even thinner by turnover and leave. While staff recognised the value of improving PEC, they were unable or unwilling to do so if this meant reducing efficiency and patient flow. The fear of disrupting service delivery was actually about interfering with systems that handled the workload. Interventions that improved the quality of care, but reduced efficiency in this resource-constrained setting, were unlikely to succeed.

While facility-level managers could see the value of better PEC, this was unlikely to be prioritised if it did not align with district goals. The need to maintain service delivery meant that appropriate staff were not sent for training or insufficient numbers of staff were trained. Those who were trained appreciated the new skill set and attempted to integrate the skills into their job, even if this was not BBCC or group education. Poor internal communication and coordination of change also hampered implementation. Health centres had not been designed with the need for group education in mind. Group education needed to be organised in a way that did not require additional visits or time in the health centre for patients.

Despite all the challenges, those patients who did benefit from improved PEC appeared to respond well, with changes in lifestyle and improved control. Although staff were motivated by these improvements in self-management and control, their overriding concern was to get through the queue and manage the workload.

### Discussion of key findings

While the evidence suggests that GREAT for diabetes and BBCC are effective interventions to improve the quality of care and outcomes for people with NCDs,^9,19,20,21^ the implementation of these in public sector primary care was far from straightforward. The core issues appeared to be with the organisational environment and culture.

Over the last few years, the health budget has decreased in real terms in the Western Cape and the health system has looked to improve efficiencies to handle the constraints.^22^ There are many signs that the real focus of service delivery is not so much on quality but on efficiency and being able to cope with high patient numbers and demands. There are many studies demonstrating high levels of burnout in primary care staff, which is a sign of high pressure and workload.^23,24^ There are indicators that clinical inertia is high and staff feel unable to take extra time to intensify treatment, initiate insulin or provide PEC.^13^ Interventions that require additional tasks or energy are avoided.

Many of the factors were related to the staff, and in this highly pressurised environment, it may be difficult to send staff away for training. Alternative models of high-frequency and low-intensity training may be better.^25^ It was clear that it was difficult to release enough staff and the right staff to attend such training. Retention of trained staff was also an issue and many organisational factors are related to high turnover of staff. These include adequate remuneration, appropriate workload, opportunities for professional development and career progression, adequate infrastructure and resources, effective management and support from specialists, as well as the ability to refer patients.^26^ Rotation of staff between different units in the health centre also reduced the availability of those who were trained. Although health promotion officers were the original target of training for group sessions, dieticians really seemed to champion this, may be because they saw the need so clearly in diabetes.

Currently, innovations for chronic disease management are a district priority, but the focus is on telehealth, appointment systems, delivery of medication and transport. Nevertheless, at the sub-district level, the Khayelitsha-Eastern sub-districts have taken a decision to scale-up GREAT for diabetes at all primary care facilities, and in Northern-Tygerberg, where the one study site was located, they have also decided to scale this up. The Department of Health has signed an MOU with Stellenbosch University to print the materials, and three dieticians employed by the Department are able to train the facilitators (one from this project).

Not sharing information was previously identified as the foremost, albeit negative, value experienced by staff in the metropolitan health services.^27^ The findings here suggest that poor sharing of information between staff within the health centres contributed to the difficulty with implementing changes. The need to provide services and handle the workload may have made it difficult to find time for regular meetings. The use of digital technology, such as WhatsApp groups, appeared to be a feasible way of improving communication. The value of such applications to communicate and coordinate was also seen later during the COVID-19 pandemic.^28^

This project was truncated by the onset of the COVID-19 pandemic, which prevented the collection of more data and further implementation of PEC in year-2. It was intended to also develop group sessions for hypertension, asthma and COPD. The materials for asthma and COPD were piloted, but not implemented, while those for hypertension, were created, but not yet piloted. The COVID-19 pandemic also led to a number of digital and technological innovations to support PEC. A GREAT for diabetes WhatsApp Chatbot took the content of the education and provided it as 16 brief audio messages to people with diabetes.^29^ This innovation has the potential to reduce workload in the facility as patients can listen to the whole programme in their own time. However, there is no real opportunity for interaction or learning from a group. The project also experimented with offering group sessions virtually via tablets and Zoom technology, and this will be reported on separately.

### Strengths and limitations

Ownership of the inquiry process is a key aspect of co-operative inquiry and the ability to explore different actions and opportunities.^15^ The researcher not only coordinated the CIGs but also brought the GREAT for diabetes and BBCC models to the table. This may have inhibited the CIGs from exploring or experimenting with other ways of implementing PEC that could have been more feasible to implement.

The facility managers and patients were not included in the qualitative interviewing because the onset of COVID-19 precluded further data collection. Two years later, as the emergency measures were withdrawn, it did not make sense to attempt further data collection. The viewpoint of the operational managers was included in the reports from the CIGs and the healthcare workers provided useful data in the FGIs.

### Implications

The following implications are identified from the findings:

- Implementing comprehensive PEC will require strong support from policymakers and implementers at subnational and district levels. Although national policy articulates the need for this, prioritisation needs to resonate at lower levels of the health system, particularly with facility managers.
- There is a need for more coordinated action at the facility level. Organising training is relatively easy, compared to the complexity of change within the organisation of services and model of care. Consistently sharing information with all staff and setting up good communication and feedback loops within the facility would help to support implementation.
- There was a tension, between the short-term need to manage the workload on a daily basis, with the middle to longer-term benefits of having better-controlled patients, who might utilise the facility less. To overcome the short-term imperatives, it may be necessary to create extra capacity to support PEC or to clearly prioritise PEC in the job descriptions of existing staff, such as health promotion officers and dieticians.
- The challenge of space for group sessions could be overcome by the use of local community facilities, but this may come with a requirement for payment. The refurbishment of the current or design of future primary care facilities should include the need for group work with patients.
- Care needs to be taken to train the appropriate staff in PEC and to ensure that a sufficient critical mass of staff are trained. Alternative methods of training online or via high-frequency and low-intensity sessions at the facility should be explored. Those not involved in PEC directly may still benefit from training in more person-centred communication skills. After training, there needs to be ongoing support with constructive feedback on the application of skills in the workplace.
- Group sessions and the sequence of sessions need to be integrated with appointment systems and patient flow so that patients do not make extra visits or take extra time, which would act as a disincentive to engage with PEC.
- If even BBCC is too long to incorporate into consultations, then it may be possible to split the process between the clinician and a lay counsellor or health promotion officer. This was successfully done for BBCC in the antenatal context with smoking cessation.^8^

## Conclusion

A number of lessons were learnt on the implementation of GREAT for diabetes and BBCC at two primary care facilities. Lessons related to the training of staff, organisational environment and the organisation of group education in the facilities. Those who had exposure to more PEC appeared to benefit in terms of knowledge, lifestyle change and improved control. Implementation of a more comprehensive PEC requires: prioritisation by managers at the district and facility levels, more coordinated action and communication in the facility, more capacity for PEC among the staff, mechanisms to create space for group work, training of the appropriate people in sufficient numbers, integration of group sessions with appointment systems and patient flow, and ways of making BBCC even briefer for clinicians.
